# Relative Survival of Peritoneal Dialysis and Haemodialysis Patients: Effect of Cohort and Mode of Dialysis Initiation

**DOI:** 10.1371/journal.pone.0090119

**Published:** 2014-03-10

**Authors:** James G. Heaf, Sonja Wehberg

**Affiliations:** 1 Department of Nephrology B, Copenhagen University Hospital at Herlev, Herlev, Denmark; 2 Department of Epidemiology, Odense University Hospital, Odense, Denmark; University of Sao Paulo Medical School, Brazil

## Abstract

**Introduction:**

Epidemiological studies consistently show an initial survival advantage for PD patients compared to HD. It has recently been suggested that this is due to the fact that many HD patients are referred late, and start dialysis on an acute, in-patient basis. The present study was performed to investigate (1) whether, and if so, how, PD and HD prognosis had changed in recent years, (2) whether a potential survival advantage of PD versus HD is constant over dialysis duration, and (3) whether differences in prognosis could be explained by patient age, renal diagnosis of diabetic nephropathy, or mode of dialysis initiation.

**Patients and Methods:**

12095 patients starting dialysis therapy between 1990 and 2010 in Denmark were studied. Prognosis was assessed according to initial dialysis modality on an intention-to-treat basis, censored for transplantation. Results were adjusted for age, sex, renal diagnosis, Charlson Comorbidity Index (CCI), and mode of dialysis initiation.

**Results:**

Overall adjusted prognosis improved by 34% (HD 30%, PD 42%). PD prognosis relative to HD improved, and was 16% better at the end of the period. Final PD prognosis improved consistently from 1990–99 to 2000–10 in all subgroups. PD was associated with a significant initial survival advantage, both overall and for all subgroups For the latter cohort, overall PD prognosis was better than HD for the first 4 years, after which it was insignificantly worse. The initial survival advantage was also present in a subgroup analysis of patients with early & routine ESRD initiation.

**Conclusions:**

Dialysis survival has increased during the past 20 years. PD survival since 2000 has been better than HD, overall and for all subgroups. The difference in survival is not explained by mode of dialysis initiation.

## Introduction

The relative survival of end stage renal disease (ESRD) patients treated with peritoneal dialysis (PD) and hemodialysis (HD) has received much epidemiological interest in recent years [1]–[25]. Most studies [1], [2], [5], [8]–[11], [14], [15], [20], [22], [25] show a relative survival advantage for patients receiving PD lasting 1–2 years after dialysis initiation. Results in the long term differ, some showing identical survival [1], [20], [22], [25] and others [4], [6]–[10], [15], [19] showing poorer survival on PD.

This consensus has recently been challenged. The ANZA [19] study showed poorer survival for PD patients than a comparison group of center-HD and home-HD patients, Another paper by Quinn et al. [22] studied a subgroup of patients with early and routine referral to a nephrologist, i.e. the group that is most likely to have made an informed choice between the two therapies, and not have it forced upon them. In this group there was no survival difference between the two groups. The suspicion arises that the initial survival disadvantage of HD patients could be due to mode of ESRD initiation, with associated acute morbidity, that may be unrecorded in registry studies.

The Danish Nephrology Registry (DNR) is a prospective, national, incident registry of all patients receiving active ESRD therapy. It was established on 1.1.1990, and has a data completeness of >99% [26]. The present study was performed to answer the following questions:

1. Has there been any change in the relative survival of PD versus HD since 1990?
2. Is the (potential) survival advantage of PD versus HD constant over dialysis duration?
3. How is PD prognosis in subgroups of patients with diabetic nephropathy, patients older than 65 years, and patients with an early (>90 days before ESRD) and routine ESRD initiation.

## Patients and Methods

### Patients

All patients resident in Denmark, and thus possessing a national identity number, starting active treatment with dialysis for ESRD between 1.1.1990 and 31.12.2010 were included in the study. Patients receiving a preemptive transplant were not included. Data were extracted from the following databases:

1. The Danish Nephrology Registry (DNR) contains data from all patients starting active treatment in Denmark. A patient is regarded as having ESRD if (a) the nephrologist considers him/her to have ESRD on the day of first active treatment or later; (b) a renal transplant is performed; (c) there is some doubt regarding the reversibility of the uraemia (e.g. crescentic glomerulonephritis, acute tubulointerstitial nephropathy), but the patient has received at least 90 days of dialysis. Patients were included if (a) their first ESRD therapy was between 1.1.1990 and 31.12.2010; (b) their first ESRD therapy was PD or HD. The following data was extracted: patient age, sex, renal diagnosis, initial therapy (PD/HD), therapy at 90 days, and all changes of therapy. Renal diagnosis was classified as unknown, glomerulonephritis, chronic interstitial nephritis (including chronic pyelonephritis and post-renal uremia), polycystic kidneys, hypertensive nephropathy, Type 1 diabetic nephropathy, type 2 diabetic nephropathy, renal cancer, myeloma, amyloidosis, systemic glomerulonephritis, vasculitis, hæmolytic uremic syndrome/thrombotic thrombocytopenic purpura (HUS/TTP) and other. This is a multicenter study covering all 15 dialysis centres in the country and their satellite units.
2. Discharge diagnoses for all admissions to public hospitals in Denmark between 1977–2010 were extracted from the National Patient Registry (LPR). A modified Charlson Comorbidity Index (CCI) [27] at ESRD was calculated, removing the contribution of the original categories based on diabetes and renal disease from the score calculation. Our CCI takes into account the presence of the following comorbid conditions: previous myocardial infarction (AMI), cardiac insufficiency, peripheral atherosclerosis, cerebrovascular disease, dementia, chronic lung disease, collagenosis, stomach ulcer, liver disease, hemiplegia, cancer, leukemia, lymphoma, liver failure, metastatic disease, AIDS [28]. Since hospital care is public in Denmark, the coverage of this database is essentially 100% [29], [30].
3. Details of patient referral were extracted from LPR. The date of first consultation at a specialist nephrology department was registered. Referrals were classified as early if the first consultation was >90 days before ESRD and late if it was <91 days. Therapy initiation was acute if the first ESRD therapy was as an in-patient and routine if it was as an out-patient. Patients were grouped as either Early & Routine (E&R), Late & Acute (L&A), and other. The LPR has registered referral dates and consultation dates for all in-patient and out-patient referrals and has been comprehensive since 1992. Data for the years 1990–1991 was incomplete.

### Methods and statistical analysis

Since most modality changes are from PD to HD, and are associated with increased mortality after change, an “as treated” model is unsuitable for this subject. Patient survival was therefore assessed on an intention-to-treat basis - only the first dialysis course was evaluated - using Cox regression models with robust variance estimation as a safeguard against misspecification. All patients were followed until either death, renal transplant or lost-to-follow-up (LTF) (censoring date 1.1.2011). Model 1 included first dialysis modality (PD, HD), cohort (according to date of dialysis initiation: 1990–1994, 1995–1999, 2000–2004, 2005–2010) patient age at date of dialysis initiation (categorized into 0-, 60-, 70- and 80-), sex, renal diagnosis, CCI (categorized into 0, 1–2, > = 3) and type of ESRD initiation (early & routine, late & acute, other). For model 2, age was substituted by the binary age > = 65 years (yes/no), and renal diagnosis was substituted by binary DM diagnosis (yes/no), i.e. patients with and without diabetic nephropathy.

To explore the development of relative survival PD/HD Prior over cohorts, an interaction term for cohort and dialysis modality was subsequently added to model 2.

To study the effect of dialysis duration (since date of dialysis initiation) on the estimated relative survival PD versus HD, a piecewise Cox regression analysis was fitted independently for the following time periods: 0–6, 6–12, 12–18, 18–24, 24–36, 36–48 and >48 months, adjusted for covariates as in model 2 but stratified for cohort (1990–99, 2000–10). Additionally, the time-dependent hazard ratio for PD versus HD based on the scaled Schoenfeld residuals [31] was estimated. We used this method by Grambsch & Therneau (which originally was proposed to check for proportional hazards) to visualize the time-dependency of the hazard ratio. The curves are generated based on Schoenfeld residuals from the (multivariate/adjusted) Cox models separately for each cohort and subgroup. The curves employ a simple Lowess smoother with bandwidth .8.

Subgroup analyses for the last analysis were performed for 1. DM diagnosis (yes), 2. age > = 65 years, 3. patients older than 65 years and with diabetic nephropathy and 4. patients with an early & routine initiation.

A supplementary analysis was performed for those patients who were still on dialysis after 90 days, where the study period started 90 days after ESRD, and the patients were classified according to their dialysis modality on that date. Type of ESRD initiation was not considered in this analysis.

The proportional hazard assumption is violated for modality in the first model, but this is not a major problem. The first model estimates the average hazard ratio over time (since the estimated hazard ratio based on a Cox model is shown to be fairly close to the exact calculation of the average hazard ratio over time). Thus, the true underlying proportional hazard ratio is not measured, just the overall/average effect. The next step was to fit piecewise models, and the last was to show time-dependent hazard ratios. This last method was originally proposed as a model check (violation of proportional hazards) but can conveniently be applied to visualize time-dependency.

Since there was a tendency over cohorts for “increased superiority”, a cohort x modality effect analysis was performed to investigate whether there were any significant effects.

Reported *p*-values are based on Wald tests. All analyses were done using Stata (StataCorp 2011. Stata Statistical Software: Release 12. College Station TX: StataCorp LP).

## Results

The patient characteristics are shown in Table 1. Patients treated with PD were generally younger, with a lower Comorbidity and a more planned ESRD initiation. Type 1 diabetic nephropathy was more prevalent in PD patients and Type 2 less. Average age increased significantly by 10.6 years for HD patients and 5.3 years for PD patients. Over cohorts, mean age ±SD increased from 55.0±15.8 in the cohort 1990–94 (HD: 55.3±16.5; PD: 54.4.±16.6) to 64.0±15.8 in the cohort 2005–10 (HD: 65.9±14.8; PD: 59.7±16.9). Mean CCI rose from 0.9±1.4 in 1990–94 to 1.6±1.8 in 2005–2010. This was not just due to increasing age. The CCI for patients aged 60–69-years rose from 1.3±1.5 to 1.8±1.9, 70–79 years from 1.2±1.6 to 2.1±1.9 and 80+ years from 0.7±1.2 to 2.1±1.7.

**Table 1:** Patient characteristics N (%) and hazard ratios (HR) together with 95%-confidence intervals (CI) for factors.

Factor				Model 1: HR (CI)	Model 2: HR (CI)
Dialysis modality^1^	HD	PD	All		
	8273 (68)	3822 (32)	12095 (100)	.89 (.84–.93)	.87 (.83–.92)**
**Cohort**				**	**
1990–94	1099 (13)	748 (20)	1847 (15)	1 (ref)	1 (ref)
1995–99	1877 (23)	792 (21)	2669 (22)	.85 (.79–.91)**	.86 (.80–.93)*
2000–04	2482 (30)	1022 (27)	3504 (29)	.7 (.65–.75)**	.76 (.70–.81)**
2005–10	2815 (34)	1260 (33)	4075 (34)	.59 (.54–.64)**	.66 (.61–.72)**
**Age**				**	
0–59	2961 (36)	1962 (51)	4923 (41)	1 (ref)	
60–69	2158 (26)	975 (26)	3133 (26)	1.88 (1.76–2.01)**	
70–79	2337 (28)	697 (18)	3034 (25)	2.7 (2.52–2.89)**	
> = 80	817 (10)	188 (5)	1005 (8)	3.62 (3.3–3.97)**	
**Age> = 65**	4335 (52)	1344 (35)	5679 (47)		2.09 (1.99–2.20)**
**Female Sex**	3045 (37)	1449 (38)	4494 (37)	.98 (.94–1.03)	.99 (.94–1.04)
**Renal Diagnosis**				**	
Unknown	1931 (23)	852 (22)	2783 (23)	1 (ref)	
Glomerulonephritis (GN)	846 (10)	590 (15)	1436 (12)	.76 (.69–.84)**	
Chronic interstitial (CIN)	996 (12)	390 (10)	1386 (12)	.92 (.85–1)	
Polycystic	504 (6)	356 (9)	860 (7)	.73 (.66–.82)**	
Hypertensive	961 (12)	430 (11)	1391 (11)	.99 (.91–1.07)	
Type 1 DM	824 (10)	632 (17)	1456 (12)	1.57 (1.44–1.71)**	
Type 2 DM	998 (12)	284 (7)	1282 (11)	1.41 (1.30–1.52)**	
Renal Cancer	136 (2)	19 (0.5)	155 (1)	1.13 (.90–1.42)	
Myeloma	207 (3)	32 (0.8)	239 (2)	2.08 (1.75–2.47)**	
Amyloidosis	71 (0.9)	41 (1)	112 (0.9)	2.11 (1.64–2.73)**	
Systemic GN	97 (1)	52 (1)	149 (1)	.98 (.76–1.26)	
Vasculitis	377 (5)	70 (2)	447 (4)	.83 (.72–.96)*	
HUS/TTP^2^	40 (0.5)	15 (0.4)	55 (0.5)	.49 (.29–.81)*	
Other	285 (3)	59 (2)	344 (3)	.97 (.83–1.13)	
**DM diagnosis**	1822 (22)	916 (24)	2738 (23)		1.51 (1.43–1.6)**
**Comorbidity**				**	**
0	2837 (34)	1941 (51)	4778 (40)	1 (ref)	1 (ref)
1–2	3623 (44)	1423 (37)	5046 (42)	1.6 (1.51–1.69)**	1.69 (1.6–1.79)**
>2	1813 (22)	458 (12)	2271 (19)	2.3 (2.14–2.46)**	2.52 (2.35–2.7)**
**ESRD Initiation**				**	**
Early & Routine	2145 (26)	1419 (37)	3564 (29)	.91 (.86–.96)*	.89 (.84–.94)**
Late & Acute	2138 (26)	648 (17)	2786 (23)	1.1 (1.03–1.16)*	1.12 (1.05–1.19)**
Other	3990 (48)	1755 (46)	5745 (47)	1 (ref)	1 (ref)

Corresponding significant *p*-values are presented as.

*: *p*<.05;

**:*p*<.001.

For definition of models see methods section.

1 :HD is the reference category;

2 : Hemolytic Uremic Syndrome/ThrombocoticThrombocytopenic Purpura.

Improvements in referral pattern were seen during the period of observation. L&A initiations fell from 37 to 16% and E&R rose from 10 to 43%.

The hazard ratios for the factors analyzed are shown in Table 1. All factors except for (female) sex had a highly significant influence on mortality in model 2. The unadjusted prognosis according to cohort and initial dialysis modality is shown in Table 2, and the adjusted in Table 3. Overall, there was a significant improvement in patient prognosis during the period of observation (HR for cohort, Table 1). Adjusted mortality fell overall by 34%. Estimated relative mortality of PD versus HD improved from .95 in 1990–94 to .8 in 2005–10 (Table 3, based on model 2 with an additional interaction term between cohort and dialysis modality).

**Table 2:** Prognosis according to cohort and modality.

Within one year:														
Cohort	Modality	N	Dead without change of modality (within 1 year)		LTF^1^ without change		Transplantation		Change of modality, later death		Change without subsequent death		Unchanged after 1 year	
			N	%	N	%	N	%	N	%	N	%	N	%
90-94	HD	1099	221	20.1	0	0	112	10.2	18	1.6	145	13.2	603	54.9
95-99		1877	446	23.8	2	.1	92	4.9	19	1	172	9.2	1146	61.1
00-04		2482	590	23.8	1	0	71	2.9	29	1.2	138	5.6	1653	66.6
05-10		2815	557	19.8	348	12.4	63	2.2	22	.8	160	5.7	1665	59.1
90-94	PD	748	59	7.9	0	0	103	13.8	28	3.7	121	16.2	437	58.4
95-99		792	66	8.3	0	0	82	10.4	34	4.3	107	13.5	503	63.5
00-04		1022	81	7.9	0	0	78	7.6	26	2.5	156	15.3	681	66.6
05-10		1260	98	7.8	154	12.2	117	9.3	25	2	174	13.8	692	54.9
Within 5 years														
90-94	HD	1099	529	48.1	0	0	231	21	85	7.7	99	9	155	14.1
95-99		1877	1048	55.8	2	.1	256	13.6	103	5.5	112	6	356	19
00-04		2482	1512	60.9	1	0	221	8.9	94	3.8	101	4.1	553	22.3
05-10		2815	1163	41.3	1157	41.1	165	5.9	70	2.5	136	4.8	124	4.4
90-94	PD	748	231	30.9	0	0	194	25.9	137	18.3	134	17.9	52	7
95-99		792	206	26	0	0	174	22	175	22.1	154	19.4	83	10.5
00-04		1022	285	27.9	0	0	231	22.6	161	15.8	219	21.4	126	12.3
05-10		1260	244	19.4	394	31.3	226	17.9	114	9	256	20.3	26	2.1
Within 10 years														
90-94	HD	1099	625	56.9	0	0	247	22.5	107	9.7	79	7.2	41	3.7
95-99		1877	1259	67.1	2	.1	296	15.8	142	7.6	79	4.2	99	5.3
00-04		2482	1766	71.2	216	8.7	259	10.4	127	5.1	75	3	39	1.6
05-10		2815	1177	41.8	1262	44.8	169	6	70	2.5	137	4.9		
90-94	PD	748	254	34	0	0	196	26.2	193	25.8	99	13.2	6	.8
95-99		792	238	30.1	0	0	184	23.2	249	31.4	112	14.1	9	1.1
00-04		1022	331	32.4	37	3.6	238	23.3	240	23.5	173	16.9	3	.3
05-10		1260	246	19.5	415	32.9	227	18	116	9.2	256	20.3		

1 : Lost to Follow-up.

**Table 3:** Cohort and mortality.

Cohort	HD	PD	Relative Mortality PD/HD
1990–94	1 (ref)	.95 (.85–1.06)	.95 (.85–1.06)
1995–1999	.88 (.8–.97)	.8 (.72–.89)	.9 (.82–1)
2000–04	.79 (.72–.86)	.66 (.59–.74)	.84 (.77–.92)
2005–10	.7 (.63–.77)	.56 (.49–.63)	.80 (.71–.89)

Adjusted hazard ratio (CI) based on a Cox regression model with interaction term between dialysis modality and cohort. Overall hazard ratio for PD versus HD was .8 (.71–.89). Overall p-value for interaction term was 0.11 (not significant).

**Figure 1:**
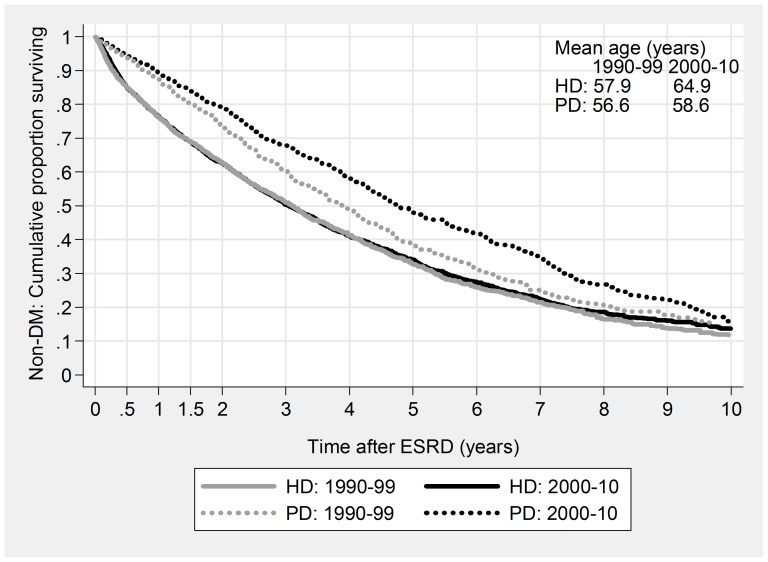
Patient Survival based on Kaplan-Meier curves, stratified for Modality and Cohort. Non-DM.

**Figure 2:**
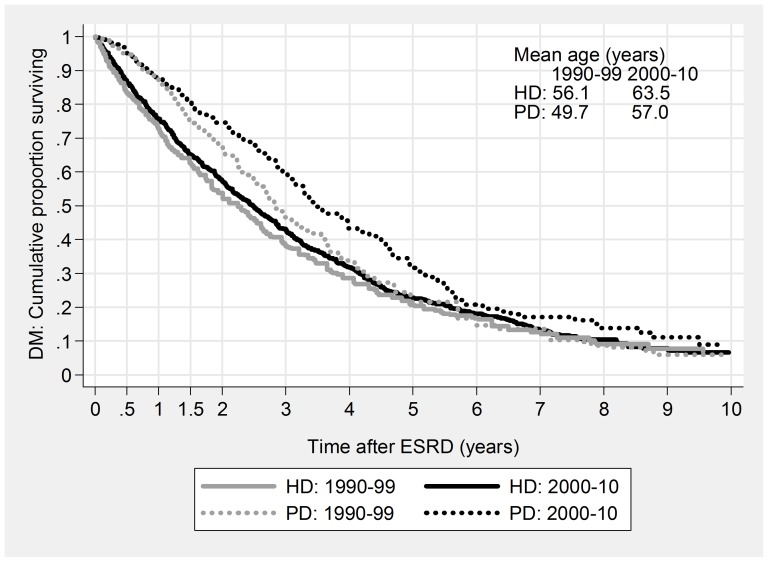
Patient Survival based on Kaplan-Meier curves, stratified for Modality and Cohort. DM.

For HD patients improvement consisted of largely unchanged mortality despite rapidly increasing age and morbidity (Figs. 1 & 2), while for PD patients non-DM median survival increased by 10 months from 1990–99 to 2000–10, while DM survival increased by 8 months, despite a seven-year increase in average age. For some subgroups, the PD improvement was dramatic: 4-year mortality for young, non-DM patients fell from 47% to 23%, and two-year mortality for elderly diabetics from 74% to 39% (Table 4).

**Table 4:** Patient mortality according to modality, age and diabetic status.

			Two-year Mortality (%)				Four-year Mortality (%)			
Modality	Diagnosis	Age (yrs)	90-94	95-99	00-04	05-10	90-94	95-99	00-04	05-10
PD	NonDM	<65	18	16	10	11	47	30	23	23
		> = 65	41	40	37	33	69	67	63	60
	DM	<65	34	22	19	21	71	53	48	46
		> = 65	74	39	36	39	100	79	74	74
HD	NonDM	<65	24	25	25	23	44	42	41	36
		> = 65	55	49	48	44	76	73	70	68
	DM	<65	43	42	31	31	64	64	56	57
		> = 65	51	60	59	49	89	84	83	74

**Figure 3:**
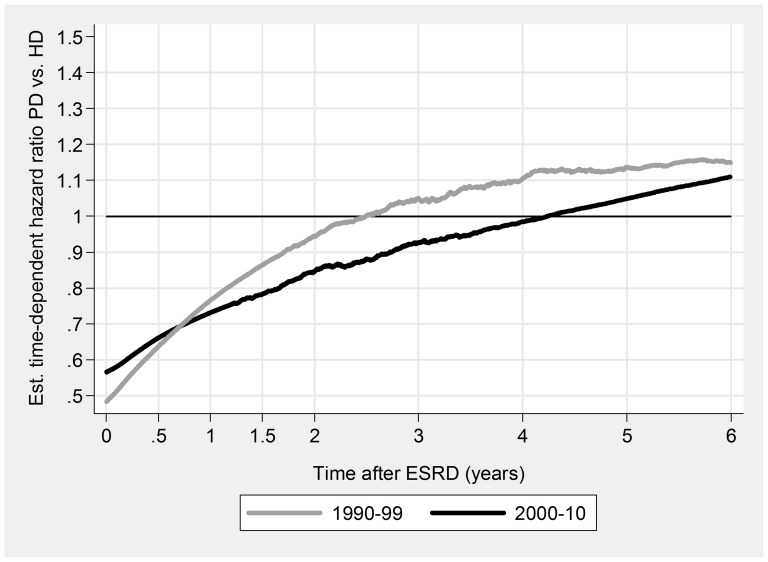
Relationship of relative mortality risk PD/HD to dialysis duration. 1990–99 versus 2000–10.

**Figure 4:**
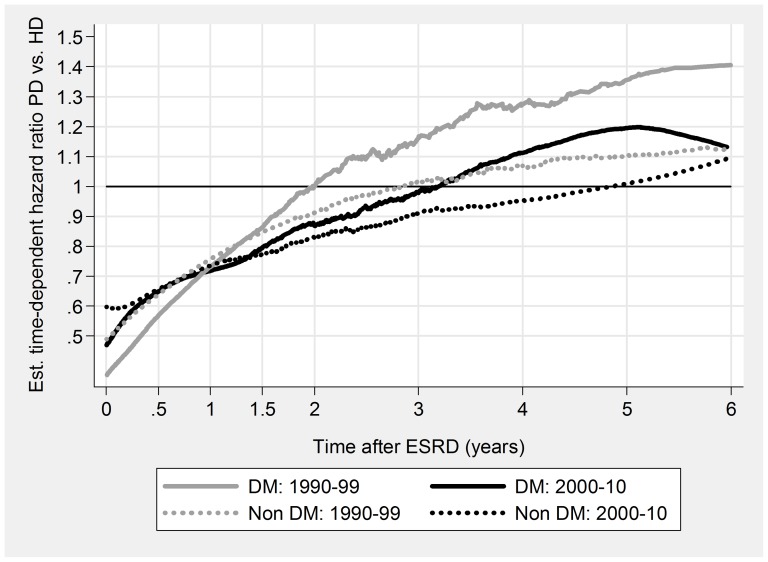
Relationship of relative mortality risk PD/HD to dialysis duration. DM versus Non-DM.

**Figure 5:**
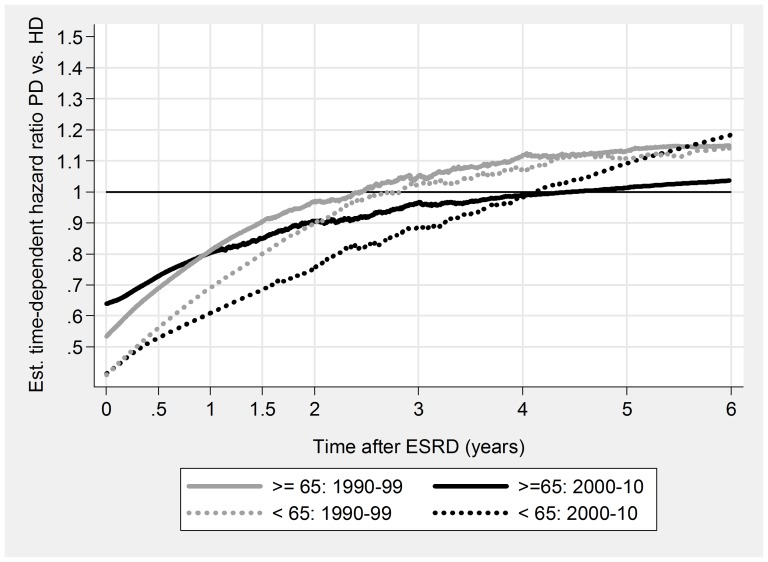
Relationship of relative mortality risk PD/HD to dialysis duration. <65 years versus > = 65 years.

PD was associated with a significant initial survival advantage, both overall and for all subgroups (Table 5, Figs. 3, 4, 5, & 6). Late prognosis was generally poorer for PD patients, but less so for the later cohort. Relative PD prognosis improved for all subgroups from 1990–99 to 2000–10. The “crossover point” from better to poorer PD prognosis increased from about 30 months to 52 months, and for no subgroup was less than 24 months. PD results were mostly better for non-DM patients than DM, and younger (<65 years) than older. Improvements in referral pattern were seen during the period of observation. Relative PD prognosis was better for patients with E&R initiation in the later cohort for the initial 36 months. There were no significant differences in age or CCI index between the E&R group and other patients.

**Figure 6:**
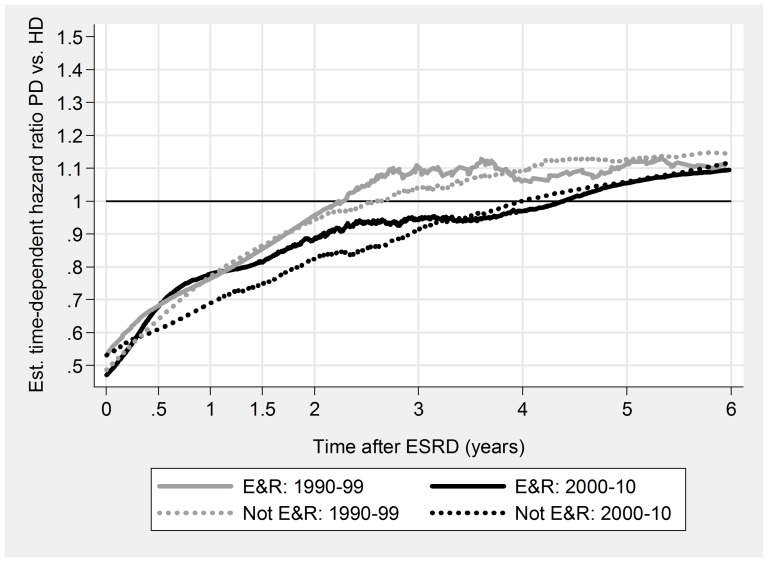
Relationship of relative mortality risk PD/HD to dialysis duration. Early & routine versus not E&R referral pattern.

**Table 5:** Relationship between relative mortality PD/HD and dialysis duration.

	All		DM		> = 65 yrs		DM and > = 65 yrs		Early & Routine	
Duration (months)	1990–99	2000–10	1990–99	2000–10	1990–99	2000–10	1990–99	2000–10	1990–99	2000–10
**0–6**	.45 (.36–.57)**	.54 (.44–.66)**	.34 (.2–.56)**	.48 (.32–.73)*	.51 (.38–.68)**	.60 (.48–.76)**	.19 (.07–.53)*	.33 (.17–.63)*	.52 (.25–1.1)	.50 (.35–.72)**
**6–12**	.79 (.63–1.00)	.69 (.56–.86)*	.78 (.49–1.25)	.76 (.52–1.11)	.84 (.61–1.15)	.78 (.60–1.01)	1.36 (.65–2.86)	.81 (.49–1.32)	.64 (.32–1.28)	.87 (.61–1.24)
**12–18**	.96 (.75–1.22)	.79 (.64–.99)*	1.01 (.66–1.55)	.73 (.49–1.08)	0.97 (.7–1.36)	.83 (.64–1.09)	1.25 (.59–2.65)	.66 (.37–1.16)	1.15 (.62–2.14)	1.12 (.8–1.57)
**18–24**	.97 (.74–1.26)	.84 (.65–1.08)	.65 (.39–1.07)	.81 (.51–1.29)	1.06 (.74–1.52)	.97 (.71–1.33)	.84 (.33–2.15)	1.04 (.54–2.01)	.73 (.39–1.38)	.66 (.43–1)
**24–36**	1.17 (.97–1.42)	.88 (.74–1.06)	1.43 (.98–2.08)	.9 (.65–1.24)	1.2 (.92–1.56)	.93 (.74–1.16)	1.65 (.87–3.13)	1.03 (.62–1.69)	1.22 (.78–1.89)	.81 (.6–1.1)
**36–48**	1.1 (.87–1.38)	1.03 (.83–1.28)	1.28 (.8–2.04)	1.32 (.91–1.9)	.94 (.67–1.32)	1.08 (.83–1.41)	1.01 (.36–2.84)	1.32 (.78–2.24)	1.18 (.65–2.14)	1.28 (.9–1.81)
**>48**	1.15 (1–1.31)*	1.12 (.96–1.32)	1.52 (1.08–2.12)*	1.11 (.81–1.53)	1.17 (.96–1.43)	1.04 (.83–1.31)	3.09 (1.38–6.90)*	1.62 (.93–2.83)	1.05 (.76–1.47)	1.05 (.8–1.38)
**Overall**	.94 (.87–1.01)	.83 (.77–.89)**	.97 (.84–1.12)	.85 (.75–.97)*	.94 (.85–1.04)	.86 (.78–.94)*	1 (.77–1.29)	.85 (.71–1.01)	.97 (.81–1.17)	.88 (.78–.99)*

Results for the 90-day analysis were broadly similar and are therefore not shown.

## Discussion

Substantial improvements in prognosis were seen for both HD and PD during the period of observation. This is in accordance with USRDS and DOPPS data [32], [33]. This is particularly remarkable, since the period has been characterized by an absence of major randomized controlled trials with a positive therapeutic result. Possible factors contributing to this general improvement include defined standards for adequate dialysis, increased attention to calcium, phosphate and PTH control, better anemia control, better preparation of dialysis initiation, and a tendency towards earlier dialysis initiation. Furthemore, advances in non-nephrological areas, such as reduced tobacco consumption, improved treatment of cardiovascular disease will also have contributed. It is possible that some of the improved prognosis is a statistical artifact, due to increased registration of comorbidity over time.

A trend to improvement in the relative prognosis of PD patients compared to HD patients were also seen, despite employing a conservative intention-to-treat analysis (ITT) strategy where the “true” group differences tend to be under- rather than overestimated. This is in accordance with previous studies [15], [19], [23], [25]. Substantial changes in the practice of PD occurred during the period. The observation of an increased mortality of patients with fast peritoneal transport [34] and the observation that automated PD (APD) seems to solve the problem [35]–[37] suggests that a major cause of PD mortality during the nineties was due to inadequate ultrafiltration secondary to rapid dissipation of the glucose osmotic gradient in fast transporters, with consequent overhydration, hypertension, pulmonary edema and death. Better fluid control using icodextrin and APD seems to have solved the problem of fast transport [36], [38]. Icodextrin became available in Denmark from 1999 and the use of APD rose rapidly from 3% in 1990 to 30% in 2000 and 66% in 2008 [39]. Biocompatible PD fluids became available in 2000, and have recently been demonstrated to preserve residual renal function (RRF), peritoneal membrane function and reduce the incidence of peritonitis and possibly other infections [40], [41]. The possible contribution of a reduction in peritonitis frequency in this study is unknown. Peritonitis frequency registration was first introduced in 2000, and has since showed a moderate improvement from 1/25 patient months to 1/31 [39]. HD-specific improvements have also occurred during the period. The introduction of biocompatible membranes and the use of “floating dry weight” (whereby fluid balance is controlled by fluid restriction rather than ultrafiltration in nonoliguric HD patients) may help to preserve RRF [42], [43]. The beneficial effects on mortality of high flux dialysis and hemodiafiltration remain however unproved [44], [45]. In conclusion, the improvement in PD prognosis has been greater than HD, and therefore cohorts from the nineties can no longer be used for comparing treatment results. Furthermore, randomized, controlled trials comparing HD and PD should offer patients the full range of PD products, including biocompatible fluids, icodextrin and APD.

As mentioned in the introduction, PD is characterized by a better initial prognosis, and an equal or worse late prognosis. One possible cause is better preservation of RRF in PD [46], perhaps due to intradialytic renal dehydration and ischemia during HD [47]. Preservation of RRF is a major determinant of dialysis survival [48]. One important exception to the general pattern is the ANZA study, which showed a poorer prognosis for PD patients, albeit with considerable improvement in the latest cohort [19]. Closer perusal of these results however [49] show that the good HD prognosis was mainly related to the high home HD prevalence in this population. If these patients are excluded from the analysis, the traditional pattern reappears, with an initial PD advantage disappearing after two years. The present study also showed a better initial prognosis, and an insignificantly worse late prognosis, again, despite a conservative ITT approach. The overall prognosis since 2000 was significantly better for PD for each of the four major subgroups.

Regardless of the overall result, most studies show a better relative effect of PD in younger [1]–[3], [5], [8], [9], [11], [12], [14]–[16], [19]–[21], [25], non-diabetic [1]–[3], [5]–[11], [14]–[16], [18], [20], [21], [23], [25] patients, and patients without comorbidity [8]–[12], [16], [19]–[21], [23], [24]. The present study also showed a relatively better prognosis for younger and non-diabetic patients, but for no subgroup was PD worse than HD. After 2000, there was no difference between relative diabetic and non-diabetic prognosis. It has been suggested [22] that the better initial prognosis is due to differences in dialysis planning, in that PD patients are more likely to have been referred earlier and started ESRD therapy as out-patients. The present study has the advantage that ESRD planning, which was found to have a major impact on prognosis, was included in the statistical analysis. In a separate analysis of patients with early, out-patient ESRD initiation, the initial PD advantage remained. Thus, differences in ESRD planning do not explain the initial PD survival advantage in this study. This conclusion is substantially different from the Quinn study [22]. It is possible that differences in patient population, protocol design and hospital practice may explain some of the differences.

All patients in the E&R group were out-patient at the time of initiation, and had been referred early to a nephrologist. This does not, of course exclude the possibility that they were acutely sick at the time of dialysis start, just that they did not need hospital admission. Furthermore, the vast majority of these patients will have been started using an AV fistula or graft, since a central catheter always requires hospital admission in Denmark. The initial PD advantage holds for all subgroups analysed, and may therefore be a universal phenomenon. Possible causes include early loss of residual function after HD initiation [47], [50] and the detrimental effects of myocardial stunning after HD initiation.

The practical consequences of this study remain controversial. Questions may be made as to the validity of the CCI and patient referral pattern. Discharge diagnosis registration may vary between centers and over time, particularly if quality measures or reimbursement rules are based on the CCI. This is not however the case in Denmark. The CCI measures only discharge diagnoses, and will thus underestimate the true comorbidity; this underestimation is not necessarily uniform between the groups studied. Despite these reservations, both CCI and referral patient were indeed very significant markers of death. As it is an epidemiological study, no causal conclusions can be drawn. The initial PD results may be due to better preservation of RRF, or to hitherto unidentified comorbid factors. The present consensus is that dialysis patients should continue to choose modality on the basis of personal preference. Patients, in particular younger, non-diabetic patients, should be informed of the possible advantage of PD. If survival is the primary motivating factor, this group of patients should however consider home HD as an alternative. It offers better phosphate, PTH, anemia and blood pressure control, fewer dietary restrictions, and removes the dangers of the long weekend [51]. Probably as a direct consequence, patient survival is greater than conventional PD and HD [49] and on a par with cadaver renal transplantation [52].
